# Endothelial Dysfunction: Insights into Systemic Lupus Erythematosus-associated Cardiovascular Disease and Neuropsychiatric Manifestations

**DOI:** 10.1007/s12265-026-10753-z

**Published:** 2026-03-07

**Authors:** Helen M. Butler, Marie Elaine Zehntner, Justin P. Van Beusecum

**Affiliations:** 1 Division of Nephrology, Department of Medicine, Medical University of South Carolina, 30 Courtenay Dr., Gazes/Thurmond Building Rm. 302, Charleston, SC USA; 2 Ralph H. Johnson VA Healthcare System, Charleston, SC 29401 USA

**Keywords:** Endothelial dysfunction, Systemic lupus erythematosus, Cardioavscular disease, Neuropsychiatric lupus, Cognitive dysfunction

## Abstract

Cardiovascular disease (CVD) and neuropsychiatric manifestations are common in patients with Systemic Lupus Erythematosus (SLE), often sharing a vascular origin with endothelial dysfunction central to their development. The endothelium plays a critical role in regulating systemic and cerebral blood flow and influencing end-organ damage in SLE. In this review, we summarize foundational and recent studies linking vascular dysfunction to CVD and neuropsychiatric outcomes, emphasizing the roles of endothelial activation, endothelial progenitor cells, blood–brain barrier dysfunction, and vascular autoantibodies. We also highlight animal models that facilitate the study of vascular and cerebral manifestations, clarifying the interconnected contributions of endothelial health to SLE-related organ damage. Finally, we discuss emerging therapeutic strategies aimed at restoring endothelial function to improve cardiovascular and neuropsychiatric outcomes in SLE.

**Graphical Abstract:**

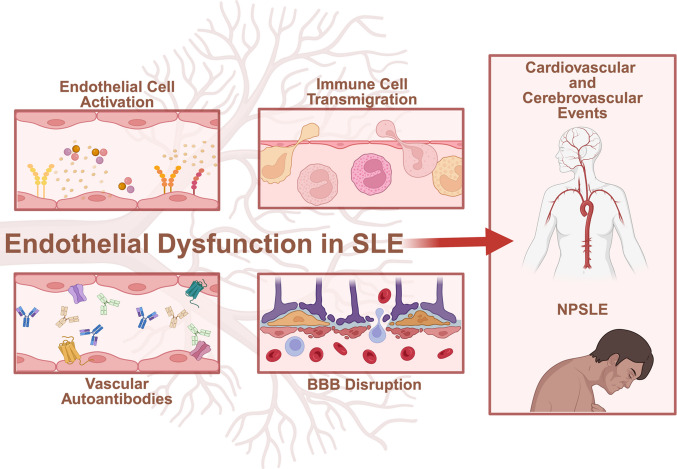

## Introduction

Systemic Lupus Erythematosus (SLE) is a chronic, multisystem autoimmune disorder characterized by immune dysregulation, hyperactive lymphocytes, complement activation, systemic inflammation, and vascular dysfunction [1]. SLE has a heterogeneous clinical presentation, with manifestations ranging from cutaneous and musculoskeletal involvement to severe complications affecting vital organs, including the cardiovascular and central nervous systems (CNS) [2]. SLE shows a significant sex bias, affecting women more often than men, and this disparity peaks during the reproductive years, pointing to a likely role for sex hormones in disease pathogenesis [3]. Women with SLE often present with a broader range of symptoms, while men may experience more severe organ involvement [4]. These differences are thought to arise from a combination of hormonal, genetic (X-linked immune-related genes), and immunological factors that influence susceptibility and progression [1]. On a global scale, SLE has an incidence of approximately 5.14 cases per 100,000 person-years and a prevalence of approximately 43.7 per 100,000 individuals, translating to roughly 3.41 million people living with the disease worldwide and approximately 0.4 million new diagnoses each year [3]. Importantly, epidemiological studies reveal that more severe manifestations of SLE are increasing in incidence [5]. Overall epidemiological patterns arise from an interplay of genetic susceptibilities, environmental exposures, inequities in healthcare access, and diagnostic opportunities.

Cardiovascular disease (CVD) is the leading cause of morbidity and mortality in SLE patients [2, 6, 7]. While improvements in SLE diagnosis and treatment have decreased mortality rates, the risk of CVD within SLE patients remains high, with different epidemiological studies estimating anywhere between a 3- to 50-fold increased risk of developing CVD events [6–11], even after accounting for traditional risk factors [12–18]. In the brain, neuropsychiatric SLE (NPSLE) can manifest as memory deficits, slowed processing, and mood disorders [19]. NPSLE affects a substantial proportion of patients and is increasingly linked to direct CNS autoimmunity, vascular injury, and microcirculatory impairment [20–28]. Cardiovascular and neuropsychiatric features in SLE can share a common vascular origin, with endothelial dysfunction and vascular inflammation central to their development.

In this review, we summarize recent findings on cardiovascular and neurovascular involvement in SLE and focus on how endothelial dysfunction serves as a mechanistic link between these organ systems. We further focus on animal models that can be used to study the manifestation of symptoms in the systemic vasculature and the brain, thereby clarifying the interconnected roles of vascular health in SLE-related organ damage. Finally, we highlight novel therapeutic opportunities that could target endothelial dysfunction to improve SLE-associated cardiovascular and neuropsychiatric outcomes.

## SLE-associated Cardiovascular Disease

Patients with SLE experience significantly higher morbidity and mortality from CVD compared to age and sex matched patient controls, with outcomes including atherosclerosis, hypertension (HTN), pulmonary arterial hypertension (PAH), and cerebrovascular events [29]. Atherosclerosis, while a main cause of CVD in the general population, has a more rapid and premature onset in SLE [30, 31]. A study of over 120 SLE patients and healthy controls found that coronary-artery calcification, an indicator for atherosclerosis, was more frequent in patients with lupus [30]. Carotid atherosclerosis [32] and arterial stiffness [33] are associated with disease duration and the Systemic Lupus Erythematosus Disease Activity Index (SLEDAI) score, respectively, suggesting that inflammation associated with autoimmunity contributes to their development in this patient population. Importantly, atherosclerosis in SLE is not accurately predicted by traditional Framingham cardiovascular risk factors [12, 13, 31].

The vascular burden of SLE extends beyond large arteries. HTN is especially prevalent, clinically relevant, and has been independently associated with cardiovascular events in SLE patients [29]. In one cohort of over 300 women, 56% of SLE patients had HTN compared to only 29% of healthy controls, with young women facing more than a threefold elevated risk [7]. Importantly, initial clinical studies indicate that HTN in SLE occurs early in the course of autoimmune disease and is not associated with advanced renal disease [34], supporting a vascular origin. Although less common than systemic HTN, PAH represents a serious cardiovascular complication of SLE [35]. A six-year follow-up study found that PAH development in patients with SLE was associated with longer SLE disease duration, organ damage, and significantly lower survival [36]. However, early diagnosis and intervention for PAH is a challenge, and close monitoring of patients with elevated risk factors is recommended [37].

Patients with SLE face an elevated risk of cerebrovascular events, including stroke, ischemia, hemorrhage, and thrombosis [38, 39]. The risk is particularly pronounced for young women during the first year following diagnosis [40]. Racial disparities also exist, with Blacks and Hispanic patients experiencing higher rates of stroke compared to other groups [41]. Key clinical features, such as increased SLE disease activity, hyperlipidemia, and HTN, have consistently associated with an increased likelihood of ischemic stroke in this patient population [42–44].

Overall, SLE is characterized by a heterogeneous spectrum of CVD complications involving the systemic and neuro- vasculature. This heterogeneity reflects the multifactorial mechanisms driving SLE pathogenesis, like endothelial dysfunction, and highlights the need for integrative approaches to understand organ-specific pathophysiology.

## Neuropsychiatric Manifestations in SLE

NPSLE can involve the central, peripheral, and autonomic nervous system and can present as one or multiple events that can precede an SLE diagnosis or occur throughout SLE disease progression. Interestingly, cognitive impairment is not always associated with disease activity [45] or overt neuropsychiatric symptoms [46, 47], suggesting the brain is compromised early in NPSLE pathophysiology and highlighting the need for early intervention in this patient population. A meta-analysis of over 5,000 SLE patients estimated that more than half of people with an SLE diagnosis will suffer from NPSLE throughout the course of the disease [48]. Like the presentation of CVD in SLE patients, NPSLE can present as a spectrum of manifestations and is unlikely to be caused by a single pathogenic mechanism. In 1999, the American College of Rheumatology (ACR) defined 19 syndromes of NPSLE, including but not limited to seizures, anxiety and mood disorders, cognitive dysfunction, and psychosis [19]. However, the diagnosis of NPSLE depends on a combination of tests, including physical examinations, brain imaging, serological tests, and neuropsychological assessments of intelligence, memory, visuospatial skills, psychomotor speed and dexterity, and mental flexibility. More recently, advanced structural MRI analysis has been proposed to augment patient clinical assessment to identify abnormalities, like white matter lesions [49, 50], in the brains of SLE patients with cognitive dysfunction [51].

## Endothelial Dysfunction in SLE: The Link between CVD and NPSLE

Pathological endothelial dysfunction is an underlying mechanism linking SLE to CVD and neuropsychiatric complications. A recent systematic review and meta-analysis, including 943 SLE patients and 644 healthy controls, confirmed these findings, in which SLE patients consistently demonstrated impaired flow-mediated dilation and increased peripheral arterial stiffness, both established surrogates of CVD risk [18]. Importantly, imaging and endothelium-dependent flow-mediated dilation studies show that patients with SLE have impaired endothelial function relative to sex- and age-matched controls, independent of traditional CVD risk factors [14–17]. In NPSLE, extensive damage to the blood–brain barrier (BBB), which is composed in part of brain microvascular endothelial cells, has been linked to cognitive impairment mediated by alterations in brain functional connectivity [52]. Here, we highlight the spectrum of endothelial dysfunction in SLE-associated cardiovascular disease and neuropsychiatric manifestations.

### Biomarkers and Peptides of Endothelial Involvement in SLE

The search for in vivo biomarkers of endothelial injury and organ dysfunction in SLE remains ongoing, offering critical insight into clinical disease progression. Many candidate markers are endothelial-derived proteins and molecules that regulate vascular immune responses. Elevated levels of soluble vascular cell adhesion molecule 1 (VCAM-1) and intracellular adhesion molecule 1 (ICAM-1) during SLE flares correlate with proteinuria, serum creatinine, anti-double-stranded DNA (anti-dsDNA) antibodies, leukocyte infiltration, and kidney fibrinoid necrosis, solidifying these adhesion molecules as early indicators of endothelial activation and flare onset [53]. VCAM-1 can also facilitate T-cell transmigration across the BBB and is upregulated on CNS endothelial cells in post-mortem cases of NPSLE [20]. Importantly, genetic lupus-prone murine models (i.e., NZB/W F1, MRL/lpr, and B6.Nba2 mice) recapitulate this increase in vascular adhesion molecules [54–56], supporting vascular and endothelial activation as a mechanism for leukocyte infiltration into tissues in SLE-associated CVD and NPSLE cerebrovascular pathophysiology.

Alongside adhesion molecules, the endothelial membrane glycoprotein thrombomodulin (TM) has also been identified as a key marker of vascular injury in SLE. TM plays a dual role as a regulator of coagulation and inflammation and is closely associated with endothelial damage. Elevated TM levels correlate with SLE disease activity, indicating greater endothelial injury as the disease progresses and highlighting their potential to identify patients at risk for cardiovascular and thrombotic events [57]. Supporting this, a biomarker study of more than 70 SLE patients, stratified by SLEDAI score and systemic involvement, found that serum TM concentrations were higher in patients with active disease than in those without active disease. Levels were further elevated in systemic SLE patients with internal organ involvement, including the lungs, heart, brain, and kidney, relative to healthy controls [58].

Vascular endothelial growth factor (VEGF) represents another critical mediator of endothelial function in SLE. By binding to its primary receptors, VEGF receptors −1 and −2 (VEGFR-1/VEGFR-2), VEGF regulates the differentiation of endothelial progenitor cells, angiogenesis, and overall endothelial cell function. Excessive VEGF levels can contribute to atherosclerotic lesion formation [59]. VEGF levels are generally elevated in SLE [60–63], with increased serum soluble VEGFR-1 and certain VEGF gene polymorphisms linked to higher disease risk in specific populations [60]. Studies have shown that VEGF is higher in patients with active SLE compared to inactive disease, and in those with systemic involvement relative to healthy controls [61]. Supporting this, a meta-analysis confirmed that VEGF levels are consistently higher in patients with SLE than in controls, particularly in active disease and lupus nephritis [62]. In a study of over 280 SLE patients, VEGF was increased in patients with increased disease activity; however, there was no association with VEGF and CVD, including atherosclerosis [63]. However, a more recent clinical study has provided evidence that highlights the potential of VEGF levels in SLE to predict cardiovascular risk [64]. A study of 83 SLE patients and 20 healthy controls found no significant difference in absolute VEGF levels between SLE and control groups, but further analyses revealed important cardiovascular associations. Stratifying subjects by VEGF levels revealed a positive correlation with carotid intima-media thickness and a negative correlation with ejection fraction, suggesting that higher VEGF levels are associated with subclinical cardiovascular dysfunction [64]. Conversely, lower VEGF levels were associated with reduced risk of developing atherosclerotic plaques [64].

The potent vasoconstrictor, endothelin-1 (ET-1), is consistently elevated in the circulation of patients with SLE [65], and has been associated with renal involvement in patients with lupus nephritis [66]. Patients with active SLE disease status and/or systemic disease have increased serum concentration of ET-1 compared to those with inactive disease and healthy control subjects [58]. However, a more recent study has found that while ET-1 was increased in SLE patients, there was no significant correlation between the serum ET-1 levels and SLE organ involvement and disease activity [67]. Increased ET-1 levels in SLE patients is recapitulated in the NZB/W F1 mouse model of SLE, which demonstrates higher levels of ET-1 and ET receptor A and B (ET_A_R, ET_B_R) mRNA in the kidneys compared to control NZW mice [68]. Notably, antagonism of ET_A_R in this model reduced blood pressure and improved glomerular filtration rate in these SLE mice [69]. In vitro studies further support a role of ET-1 in endothelial dysfunction in SLE. Endothelial cells incubated with serum from SLE patients demonstrate increased ET-1 secretion relative to healthy control serum incubations [70].

### Immune Activation of the Endothelium in SLE

Immune dysregulation in SLE influences endothelial function and contributes to the pathophysiology of SLE-associated CVD and NPSLE. Immune-mediated vascular pathology can include endothelial activation, immune complex deposition, and sustained inflammatory injury [56, 71, 72]. Platelet activation and heightened type I interferon (IFN) signaling are associated with endothelial activation and impaired peripheral endothelial function in SLE patients [73], underscoring the central role of innate immune pathways in driving vascular dysfunction.

Neutrophils are effector cells that mediate endothelial injury in SLE. Increased expression of CD177 on neutrophils has been associated with disease activity in a Chinese SLE cohort [71]. Furthermore, CD177⁺ neutrophils exhibit enhanced formation of neutrophil extracellular traps (NETs), which have been shown to promote endothelial cell death and vascular dysfunction in an imiquimod-induced lupus mouse model [71]. In parallel, adaptive immune activation also contributes to vascular disease progression in SLE. β2 glycoprotein I (β2GPI) reactive T cells have been identified in SLE patients and are associated with increased subclinical atherosclerosis [74], providing a mechanistic link between autoantigen-specific T cell responses and endothelial pathology.

Cytokine-driven alterations in endothelial homeostasis further contribute to endothelial pathology in SLE. In vitro studies using primary human endothelial progenitor cells demonstrate that IL-10 negatively impacts endothelial cell differentiation, especially in the context of IFN-mediated disease like SLE, potentially impairing vascular repair mechanisms [75].

Preclinical models reinforce the immune contribution to SLE-associated CVD pathology. Microvascular inflammation, damaged vessel walls, and red blood cell extravasation are observed in the hearts of R848-treated B6.*Sle1*.*Sle2*.*Sle3* mice, whereas these features are absent in mice lacking B and T cells (Rag^*−/−*^) [72]. Moreover, transfer of splenocytes or serum from R848-treated mice induces myocardial hypertrophy and cardiac immune-cell infiltration in untreated B6.Sle1.Sle2.Sle3 recipients, indicating that lymphoid, non-lymphoid, and systemic inflammatory factors collectively drive SLE-associated CVD [72].

In NPSLE, immune-mediated endothelial dysfunction intersects with neuroimmune activation. NPSLE is associated with upregulation of adaptive immune responses, complement activation, and RNA expression signatures that are particularly pronounced during active disease [76]. Activated microglia contribute to this inflammatory milieu by producing cytokines such as IL-6 and IL-8, which can induce neuronal injury prior to overt BBB disruption [77]. Notably, a meta-analysis and RNA-seq study demonstrated significant upregulation of C–C motif chemokine ligand 2 (CCL2) in SLE [78]. In vitro data further confirm high CCL2 expression in peripheral blood dendritic cells from NPSLE patients, which has been shown to induce endothelial cell pyroptosis [78]. Collectively, these findings underscore the role of immune-driven endothelial dysfunction as a central mechanism in NPSLE pathogenesis.

### Endothelial Progenitor Cells (EPCs) in SLE-associated CVD

Circulating endothelial progenitor cells (EPCs) can help protect against CVD, especially atherosclerotic disease, through regeneration and repair of the vasculature [79]. EPCs can be classified by their extracellular markers into circulating angiogenic cells, endothelial colony-forming cells, and myeloid angiogenic cells [80]. The literature of EPC levels in SLE is mixed. It has been reported that patients with SLE have lower levels of circulating and/or functional EPCs even in clinical remission of patient disease [81–87]. This clinical finding is supported by the spontaneous NZB/W lupus murine model which demonstrates reduced number and function of EPCs [88]. Alternatively, increases in EPC number and function, as well as levels of angiogenic markers, have also been found in SLE cohorts [80, 89, 90]. Recently, studies utilizing flow cytometry have proposed that novel clusters of EPCs, rather than their overall expression, are potential biomarkers of SLE disease progression [80, 91]. Specifically, these novel CD31 + clusters are associated with SLE remission [80], organ damage defined by the Systemic Lupus International Collaborating Clinics Damage Index (SDI) [80], as well as cardiovascular disease risk [91], suggesting impaired functionality of EPCs in SLE patients.

### Blood Brain Barrier Dysfunction and Cerebral Oxygenation in NPSLE

The BBB, formed in part by brain microvascular endothelial cells, maintains a stable microenvironment for the CNS. Disruption of the BBB has been proposed as a key mechanism underlying the pathophysiology of NPSLE. Evidence supporting this mechanism comes from perfusion MRI studies in SLE patients, which demonstrate increased BBB leakage, particularly in regions such as the hippocampus [21, 22]. Extensive BBB leakage has also been associated with poorer performance on cognitive tasks [23]. In a recent study of over 100 SLE patients, nearly 25% exhibited significant BBB leakage, which correlated with cognitive impairment [24]. Notably, this leakage did not associate with 18 serological markers and circulating autoantibody levels, suggesting that BBB dysfunction may represent a pathological process independent of traditional SLE hallmarks.

Beyond barrier integrity, cerebral microcirculation and oxygen metabolism also appear to contribute to neuropsychiatric symptoms in SLE. Impaired microcirculation can disrupt oxygen delivery and the brain’s ability to extract oxygen from blood [92], a process quantified by the oxygen extraction fraction (OEF). Mapping of cerebral OEF in NPSLE, non-NPSLE, and healthy controls has revealed regional differences that associate with cognitive impairment [93]. Altered cerebral oxygenation has also been linked to mood disorders. In a small study of 23 SLE patients without known antidepressant or antipsychotic drug treatment, depression severity (assessed by the Beck’s Depression Inventory I) was associated with lower oxygenated hemoglobin during light exercise, as measured by near-infrared spectroscopy [94]. Higher depression scores in this study were also correlated with elevated VCAM-1 levels, pointing to a mechanistic link between cerebral endothelial dysfunction, impaired oxygenation, and neuropsychiatric manifestations of SLE.

### Vascular Autoantibodies

Circulating autoantibodies can also drive endothelial dysfunction and cardiovascular risk in SLE. Anti–double-stranded DNA (anti-dsDNA) antibodies, a diagnostic hallmark of SLE, fluctuate with disease activity and are associated with vascular pathology [95]. In a study of over 80 SLE patients, presence and persistence of anti-dsDNA was associated with higher CVD risk, including increased carotid intima-media thickness, impaired microvascular function, increased plasma atherogenic risk index, and a higher ApoB/ApoA ratio [96]. This association between anti-dsDNA titers and endothelial dysfunction was independent of other typical CVD risk factors, like HTN, smoking, and obesity. Utilizing co-culture in vitro studies, Patiño-Trives et al. further suggests anti-dsDNA antibodies promote leukocyte mediated endothelial dysfunction, characterized by increased expression of VCAM-1, ICAM-1, and eNOS, along with elevated reactive oxygen species (ROS) levels [96]. Increased levels of anti-phospholipid antibodies, commonly increased in SLE pathophysiology have also recently been associated with cerebral stroke and increased carotid intima-media thickness [97] as well as increased valvular disease in SLE patients [98].

Autoantibodies directed against endothelial cell structures, termed anti-endothelial cell antibodies (AECA), were first reported in human kidney biopsy samples in the 1970 s [99]. Initially thought to be nonspecific, subsequent studies have demonstrated that many of these autoantibodies are target-specific, exert functional effects, and are closely linked to disease states. Among autoimmune diseases, SLE has the largest number of detectable autoantibodies which target membrane, cytoplasmic, and cell membrane antigens across multiple tissues. AECA are detectable in an estimated 75% of SLE patients and are positively associated with disease activity, as measured by SLEDAI scores [100]. Functionally, AECA can drive both cardiovascular and cognitive manifestations of SLE through direct activation of endothelial cells. This includes upregulation of adhesion molecules like E-selectin, ICAM-1, and VCAM-1, as well as nuclear translocation of the nuclear factor-kappa B (NFkB) p65 subunit and increased secretion of inflammatory cytokines and chemokines [101–104]. For example, AECA have been associated with soluble E-selectin and VCAM-1 levels and the initial stages of vascular damage in an SLE cohort of 56 women grouped by AECA positivity [105].

AECA are strongly implicated in CVD. For example, functional autoantibodies against alpha 1-adrenergic receptors (α_1_-ARs) act as agonists by binding specific epitopes of the receptor’s extracellular loops and have been implicated in HTN [106]. Because α_1_-ARs play an important role in endothelial cell biology, these findings suggest that HTN pathogenesis may, in part, be autoimmune in origin. Similarly, autoantibodies against angiotensin II type 1 receptor (anti-AT_1_R) mimic angiotensin II signaling and contribute to both lupus nephritis and malignant HTN [107, 108]. Anti-AT_1_R and those directed against endothelin receptor A (anti-ET_A_R) are also common in systemic sclerosis and are positively associated with more severe disease states and disease-related mortality [109]. Anti- AT_1_R and anti-ET_A_R autoantibodies bind to and activate endothelial cells in vitro [109, 110], evidenced by increased cell adhesion molecule expression, ROS, and neutrophil migration through an endothelial layer [75]. In SLE, anti-ET_A_R autoantibodies are elevated in patients with PAH and have been shown to functionally accelerate disease progression [111]. Although the precise clinical relevance of vascular autoantibodies remains incompletely defined, accumulating evidence supports their role in linking anti-endothelial autoimmunity with cardiovascular complications in SLE.

Brain reactive autoantibodies are detectable in both SLE and NPSLE patients, however their frequency and circulating levels are often higher in individuals with NPSLE [112]. These autoantibodies can include neuronal associated targets such as glyceraldehyde 3-phosphate dehydrogenase (anti-GAPDH), β2-glycoprotein-1 (anti- β2GP1), and triosephosphate isomerase (anti-TPI). Notably, anti-GAPDH autoantibodies have been positively associated with elevated intracranial pressure and cerebral vascular lesions in a cohort of NPSLE patients, which are pathophysiological features that are thought to underly a substantial proportion of NPSLE symptomology [113].

Importantly, autoantibodies against endothelial cell targets have also been linked to both overall SLE disease activity and CNS involvement [25]. AECA activation of endothelial cells can disrupt the BBB and promote the development of NPSLE, however these autoantibodies can also have a direct cytotoxic effect through complement activation or induced coagulation on endothelial cells as well as augmentation of leukocyte adhesion to the endothelium in inflammation [26]. Notably, associations have been reported between AECA and psychiatric symptoms, including psychosis and depression. In one study of 51 SLE patients, AECA were present in over 60% of those with psychiatric manifestations compared with fewer than 30% of those without such symptoms [27]. Autoantibodies against non-canonical endothelial targets, like N-methyl-D-aspartate receptors (NMDARs), are associated with NPSLE as well. NMDARs, found on brain endothelial cells, function in vasodilation and BBB integrity [28]. Importantly, serum autoantibodies against NMDARs (anti-NR2) in SLE patients are linked to depression as scored by the Beck Depression Inventory II (BDI-II), but not cognitive dysfunction, including memory, attention, visuospatial, motor, and psychomotor function [114]. Therefore, the detection of AECA in the circulation of SLE patients with depression supports a biological basis for mood disorders in this patient population.

## Experimental Models of SLE with Cardiovascular and Cognitive Dysfunction

Murine models of SLE are valuable tools to investigate disease pathophysiology across multiple organs, including the heart, brain, and vasculature. Inducible models of SLE include but are not limited pristane injection or Resiquimod (R848) administration. A single injection of pristane, a naturally occurring hydrocarbon, mimics many of the clinical features of SLE, including diverse autoantibody production, systemic inflammation, and kidney involvement [115]. Progressive increases in immune complex and lipofuscin deposition in brain regions have been documented in pristane induced lupus (PIL) mice [116, 117]. Similarly, administration of R848, a Toll-like receptor 7 agonist, induces autoantibody production and cardiac pathology including increases in hemorrhagic lesions, immune cell infiltration, iron deposits, and immune complex deposition [118–120]. While other inducible murine models of SLE exist (i.e. hydralazine induced lupus and chronic graft-versus-host disease models), here we focus on inducible mouse models of SLE with documented cardiovascular involvement affecting vascular, cardiac, or cognitive function (Table 1).

**Table 1:** Cardiovascular and cognitive dysfunction in murine models of SLE

Model		Cardiovascular Dysfunction	Cognitive Dysfunction
Inducible	Resiquimod (R848)	1) Acute hemorrhagic myocarditis in CFN mice [120] 2) Left ventricular dilation and reduced systolic function in CFN mice [119]	
	Pristane	1) Increased MAP(tail cuff) [157] 2) Increased MAP (indwelling arterial catheter) [165] 3) Reduced endothelium-dependent maximal relaxation of aortic rings [157] 4) Impaired endothelium-dependent relaxation of second order mesenteric arteries [165]	1) Reduced interest in olfactory attractants [116] 2) Reduced locomotion [116] 3) Increased anxiety [116, 117] 4) Depressive behavior [116, 117] 5) Impaired spatial memory [117, 166, 167] 6) No recognition memory deficits [116] 7) No social deficits [116] 8) No motor skill deficits [116]
	Anti-NMDAR injection		1) Impaired spatial memory [168]
	Anti-ssDNA injection		1) Impaired spatial memory [169] 2) Impaired recognition memory [169] 3) No depressive behavior [169] 4) No differences in locomotion [169]
Spontaneous	NZB/W F1	1) Increased MAP (radiotelemetry) [170] 2) Increased MAP [171, 172], increased SBP [153] (indwelling arterial catheter) 3) Increased SBP (tail cuff) [123, 153] 4) Increased MAP (tail cuff) [173] 5) Reduced endothelium-dependent maximal relaxation of aortic rings [88, 153, 173] 6) Impaired endothelium-dependent relaxation of carotid arteries [170]	1) Increased anxiety [77, 174] 2) Depressive behavior [77] 3) Impaired spatial memory [175] 4) Impaired recognition memory [77] 5) Impaired discrimination learning [175, 176] 6) Impaired motor performance [77] 7) Social deficits [77]
	MRL/*lpr*	1) Reduced endothelium-dependent maximal relaxation of aortic rings [126, 177] 2) No difference in SBP (tail cuff) [177] 3) Increased aortic intima media thickness [127]	1) Anhedonia [129, 178–181] 2) Reduced locomotor activity [130, 131, 182, 183] 3) Increased anxiety [183] 4) Depressive behavior [128, 130, 183, 183, 185] 5) Impaired spatial memory [182, 185] 6) Impaired learning [176] 7) Reduced exploratory behavior [186] 8) No deficits in novel object recognition [184] 9) No deficits in spatial learning [176]
	BXSB		1) Impaired discrimination learning [175]
	B6.*Nba2*	1) Systolic and diastolic dysfunction (R848 acceleration) [56]	1) Increased anxiety [187] 2) Increased depressive behavior [187] 3) Altered fear response [135] 4) No recognition memory deficits [135, 187] 5) No sensory motor or motor skill deficits [187] 6) No change in depressive behavior [135]
	B6.*lpr*	2) Reduced endothelium-dependent maximal relaxation of aortic rings [88]	
	B6.*Mecp2*^Tg^ [1] B6.*Mecp2*-null		1) Reduced locomotion [188] 2) Increased anxiety [188] 3) Impaired recognition memory [188] 4) Intact social ability [188] 5) Motor deficits [189]
	B6.*Sle1.Sle3*		1) Anhedonia [190] 2) Reduced motor coordination [190] 3) Impaired spatial memory [190] 4) Impaired recognition memory [190]

In contrast, spontaneous genetic models develop lupus-like disease without external triggers. The NZB/W F1 mouse represents the classical SLE mouse model, exhibiting systemic autoimmunity, hemolytic anemia, proteinuria, immune complex deposition, and glomerulonephritis [121, 122]. Female NZB/W F1 mice develop earlier and more severe disease than males, reflecting the female predominance observed in patients. Cardiac pathology in NZB/W F1 include increased heart weight to body weight ratio, left ventricular wall thickness, proinflammatory cytokine levels, cardiac immune cell infiltration, myocardial complement deposition [123]. In this model, microglial activation and disruptions in neurogenesis that affect the hippocampus are signs of early NPSLE, with BBB disruption and classical interferon pathophysiological signaling occurring later in disease progression [77].

The MRL/lpr strain, homozygous for the lymphoproliferation spontaneous (*Fas*^lpr^) mutation, develops systemic autoimmunity, immune complex deposition and glomerulonephritis [124]. Although females generally exhibit worse outcomes, both sexes demonstrate robust autoimmune phenotypes. As in the patient population, the MRL/*lpr* mice display considerable heterogeneity in systemic manifestations [125]. Cardiovascular pathology includes elevated aortic VCAM-1 levels [126] and increased intima-media thickness, immune cell infiltration, cytokine expression of the aorta [127]. Cerebrovascular pathology in these mice can include increased BBB disruption [128], elevated levels of brain-reactive autoantibodies [129–131], and increased IL-6 levels in the serum and cerebral spinal fluid (CSF) [132], all of which correlate with behavioral outcomes. Interestingly, B cells and/or autoantibodies are not alone responsible for NPSLE pathogenesis in the MRL/*lpr* model, as constitutive and conditional B-cell depletion shows no improvement in BBB integrity and cognitive function, even with autoantibody depletion in the serum and CSF [133]. Also, unlike NZB/W F1 mice, MRL/*lpr* mice exhibit fewer genetic brain abnormalities, like ectopic neurons, which can confound interpretations of behavioral outcomes [134].

Beyond these classical spontaneous models, less severe models of SLE, including the B6.*Nba2*, B6.*lpr*, B6.*Mecp2*, and Sle1/Sle2/Sle3 genetic strains, are additional tools to study functional cardiovascular and cognitive outcomes in SLE. We have recently published cardiovascular pathophysiology in the B6.*Nba2* mouse model, which revealed immune complex deposition in both the heart and brain, increased cardiac perivascular fibrosis, and increased level of circulating soluble VCAM-1 [56]. The B6.*Nba2* model also demonstrates an NPSLE phenotype, including increased anxiety and fearful behavior, regardless of the presence of Toll-like Receptor 7 (TLR7) [135]. As TLRs are potentially involved in the production of anti-nuclear autoantibodies [136], these findings suggest that mechanisms beyond canonical SLE autoantibody pathophysiology drives neuroinflammation in NPSLE. Functional cardiovascular and cognitive outcomes in these SLE mouse models are describe in Table 1.

## Therapeutically Targeting Endothelial Dysfunction in SLE

A key strategy to reduce cardiovascular risk in SLE patients is targeting endothelial activation and atherosclerosis. Statins not only lower low-density lipoprotein and increase high-density lipoprotein levels, thereby limiting atherosclerotic progression, but also exert direct vascular benefits. They have been shown to improve coronary endothelial function in acute coronary syndrome patients [137], reduce arterial stiffness in rheumatoid arthritis (RA) patients [138], and improve flow mediated dilation in both RA [139] and SLE patients [140]. In a retrospective study of more than 4000 SLE patients with co-morbid hyperlipidemia, statin therapy was associated with reduced all-cause mortality and CVD events [141]. Meta-analyses further demonstrate that statin therapy reduces circulating molecules and markers of endothelial activation, including plasma ET-1 [142] and VEGF [143], across multiple randomized controlled trials. Although atorvastatin did not lower antiphospholipid antibody levels after one year of treatment in SLE patients [144], statins may mitigate the downstream effects of autoantibodies on endothelial cells. For example, in vitro treatment with fluvastatin reduced anti-phospholipid antibody-induced monocyte adhesion, as well as ICAM-1 and IL-6 expression, in human umbilical vein endothelial cells [145].

Beyond lipid lowering therapies, other pharmacologic strategies in SLE focus on modulating the renin-angiotensin and the endothelin system. Angiotensin-converting enzyme (ACE) inhibitors have been investigated for their ability to reduce vascular dysfunction and lower CVD in SLE. ACE inhibitors can reduce CVD related morbidity and mortality through both hypotensive effects and improvement of endothelial cell function, as demonstrated in RA patients [146]. More recently, Oliveira et al. evaluated the effects of ramipril on endothelial cell function in a cohort of 37 SLE patients without who lacked traditional CVD risk factors [147]. Ramipril treatment improved endothelial function, assessed by flow mediated dilation, and was associated with increased numbers of colony-forming monocytic endothelial progenitor cells from patient PBMCs, along with higher circulating VEGF levels. In parallel, endothelin receptor antagonists have emerged as a therapeutic treatment for resistant HTN and PAH, common CVD manifestations in SLE patients. Specifically, endothelin receptor antagonists have shown improvement of cardiac hemodynamics and in lowering blood pressure [148–151]. However, the use of endothelin receptor antagonists specifically in SLE patients has not been extensively studied.

In addition, agents with broader immunomodulatory and cardioprotective effects have also been investigated in SLE. Hydroxychloroquine (HCQ), a standard therapy for SLE, not only reduces disease flares but may also confer vascular benefits. Higher circulating HCQ levels have been linked to reduced levels of endothelial activation markers, including soluble VCAM-1 and ICAM-1, in SLE patients [152]. Similar effects have been demonstrated in the NZB/W F1 mouse model, wherein HCQ treatment lowered systolic blood pressure and improved endothelium-dependent vasodilatory responses to acetylcholine in thoracic aortas [153]. More recently, HCQ has also been associated with increased numbers of circulating angiogenic cells, suggesting an additional mechanism through which it may support vascular repair [86].

Beyond established pharmacologic therapies, interest has also grown in the role of lifestyle and supplemental factors in modulating endothelial dysfunction in SLE. Specifically, Vitamin D and caffeine have been investigated for their potential effects on endothelial function and vascular health. Vitamin D supplementation is commonly recommended for SLE patients, as circulating levels of vitamin D are inversely associated with disease activity [154]. In vitro studies have shown that vitamin D can increase the number and improve the migratory and proliferative abilities of EPCs from SLE patients, effects that correlate with nitric oxide production [155]. These findings suggest vitamin D supplementation may improve endothelial function in this patient population. Similarly, caffeine appears to exert a protective role on the endothelium in SLE. Higher caffeine intake is positively correlated with the percentage of circulating EPCs in SLE patients [156]. Supporting this, in vitro experiments showed that EPCs from healthy donors that were then exposed to SLE serum had improved morphology and increased colony forming units when incubated with sera from high caffeine SLE subjects compared to low caffeine subjects. Together, these observations indicate that both vitamin D and caffeine may contribute to maintaining endothelial health in SLE.

Recent in vivo studies have demonstrated that therapeutic targeting of Rho-associated coiled-coil-containing protein kinases (ROCKs) and the mammalian target of rapamycin complex 1 (mTORC1) can attenuate vascular dysfunction in SLE murine models. For example, coptisine, a ROCK inhibitor, administered intraperitoneally to pristane BALB/c mice, lowered blood pressure and improved endothelium vasodilation [157]. Similarly, an 8-week treatment of rapamycin, an mTORC1 inhibitor, in MRL/lpr and healthy control MRL/MpJ mice reduced aortic VCAM-1 levels and restored endothelium-dependent maximal relaxation of thoracic aortas to control levels [126].

Less is known about strategies that directly target endothelial cell activation in the treatment of NPSLE. Glucocorticoid (GC) pulse therapy has been shown to significantly reduce levels of brain reactive autoantibodies in the CSF of NPSLE patients, including the non-canonical endothelial cell target, NMDAR [158]. Notably, GC therapy was associated with improvements in neuropsychiatric symptoms and SLEDAI scores in this cohort. However, no significant correlations were observed between post-treatment SLEDAI scores and CSF autoantibody levels [158], suggesting that improved clinical outcomes following GC therapy is not solely driven by autoantibody burden.

Preclinical in vivo murine studies support the therapeutic relevance of targeting endothelial-leukocyte interactions and neuroinflammation in NPSLE. In the MRL/lpr mouse model, administration of small molecule cell adhesion inhibitor, K-7174, reduced the expression of endothelial cell adhesion molecules CD31 and VCAM-1 in brain microvascular endothelial cells while enhancing tight junction expression. These changes were associated with decreased BBB permeability and the attenuation of cognitive dysfunction [159]. Similarly, immunomodulation with FTY720 in MRL/lpr reduced inflammatory cytokine production and immune cell infiltration into the central nervous system, while improving BBB integrity and neuropsychiatric outcomes [160]. Systemic fingolimod treatment in MRL/lpr mice has also been shown to reduce brain leukocyte infiltration, pro-inflammatory signaling in endothelial cells, and cognitive dysfunction [161]. More recently, a preventative effect of *Cinnamomum cassia* powder has been reported in the imiquimod-induced NPSLE model in C57BL/6 J mice, where disease progression was mitigated through preservation of tight junctions, reduced BBB permeability, and improved cognitive performance [162]. Beyond direct modulation of the vasculature, ACE inhibition has shown reduced microglial activation and preserved cognition in an anti-DNA/NMDAR autoantibody-induced mouse model [163]. Collectively, these findings highlight vascular and endothelial activation as well as BBB dysfunction as modifiable drivers of NPSLE pathogenesis. However, further studies are needed to translate these findings into targeted clinical treatments for NPSLE.

## Mind the Gap: Clinical Therapies Targeting the Endothelium in NPSLE

While recent preclinical studies have advanced our understanding of endothelial involvement in SLE-associated cardiovascular disease, direct clinical investigation of endothelial dysfunction in NPSLE is limited. Currently, there are no approved therapies specifically for NPSLE, and only a single efficacy and safety trial is registered on ClinicalTrials.gov, evaluating the NMDAR antagonist EG-501.

One major challenge in advancing NPSLE therapies is the marked heterogeneity of clinical presentation. Patients may exhibit a broad spectrum of neuropsychiatric manifestations, ranging from mood disorders, anxiety, and cognitive dysfunction to more severe symptoms such as seizures and psychosis. Consequently, large SLE drug trials frequently include neuropsychiatric outcomes only as secondary or exploratory endpoints [164], limiting their ability to rigorously assess therapeutic efficacy in NPSLE. Altogether, these gaps underscore an urgent need for mechanistically informed, endothelial-focused clinical studies designed specifically for NPSLE.

## Conclusion

Multiple clinical, *in vivo*, and *in vitro* studies have advanced our understanding of the mechanisms driving endothelial dysfunction and associated cardiovascular and neurovascular complications in SLE. This review highlights key clinical investigations and murine models that elucidate how endothelial activation contributes to both SLE-related CVD and neuropsychiatric manifestations. Emerging evidence underscores the roles of endothelial activation, endothelial progenitor cells, blood–brain barrier dysfunction, and vascular autoantibodies in mediating endothelial pathology in SLE. We also emphasize recent therapeutic approaches with dual benefits, including the restoration of endothelial function. Future studies, particularly those that directly target the endothelium, are needed to further elucidate the mechanisms linking SLE to cardiovascular and neurovascular outcomes.
